# Rutin Attenuates Hepatotoxicity in High-Cholesterol-Diet-Fed Rats

**DOI:** 10.1155/2016/5436745

**Published:** 2016-04-27

**Authors:** Shakir D. AlSharari, Salim S. Al-Rejaie, Hatem M. Abuohashish, Mohamed M. Ahmed, Mohamed M. Hafez

**Affiliations:** ^1^Department of Pharmacology and Toxicology, College of Pharmacy, King Saud University, P.O. Box 2457, Riyadh 11451, Saudi Arabia; ^2^Department of Biomedical Dental Sciences, College of Dentistry, University of Dammam, Dammam 31441, Saudi Arabia

## Abstract

*Background and Objective.* High-cholesterol diet (HCD) intends to increase the oxidative stress in liver tissues inducing hepatotoxicity. Rutin is a natural flavonoid (vitamin p) which is known to have antioxidative properties. The aim of the present study was to investigate the potential effects of Rutin on hypercholesterolemia-induced hepatotoxicity in rats.* Materials and Methods.* Male Wistar rats were divided into four groups: G-I control, G-II Rutin, G-III HCD, and G-IV Rutin + HCD. The liver functions and lipid profile were used to evaluate the HCD-induced hepatotoxicity. Quantitative real time-PCR was carried out to evaluate the expression levels of genes in TGF-*β*/Smad signaling pathway.* Results.* Rutin in combination with HCD showed a significant protective effect against hepatotoxicity. HCD caused significant increase in the mRNA expression of transforming growth factor beta (*TGF-β*), Mothers Against Decapentaplegic Homolog 2 (*Smad-2*), Mothers Against Decapentaplegic Homolog 4 (*Smad-4*), Bcl-2-binding component 3 (*Bbc3*),* caspase-3*,* P53* and Interleukin-6 (*IL-6*) and decrease in the expression levels of Cyclin depended kinase inhibitor (*P21*) and Interleukin-3* (IL-3*) in hepatic cells.* Conclusion. TGF-β/Smad* signaling pathway is involved in HCD-induced hepatotoxicity and Rutin inhibits the hepatotoxicity via suppressing this pathway. Therefore, Rutin might be considered as a protective agent for hepatotoxicity.

## 1. Introduction

Nonalcoholic steatohepatitis (NASH) is a form of chronic liver disease and a part of nonalcoholic fatty liver disease (NAFLD), which may lead to cirrhosis and hepatocellular carcinoma (HCC) [1–7]. The prevalence of NAFLD has significantly increased worldwide with the increase of obesity [4, 8]. Investigating the mechanisms of NAFLD may help in finding the strategies against this disease [9]. Oxidative stress induced by high-cholesterol diet can mediate a variety of cellular responses leading to diverse outcomes such as apoptosis [10, 11], which is involved in NASH causation [12].

Apoptosis occurs in many human liver disorders [13, 14] and can trigger cell repair, inflammation, regeneration, and fibrosis [15]. Liver fibrosis may result in cirrhosis and end-stage liver disease [16, 17]. The uncontrolled hepatocyte apoptosis may be a central mechanism, triggering liver fibrogenesis [18]. The hepatocyte is specific genetic disruption of the antiapoptotic member of the Bcl-2 family Bcl-xL resulting in hepatocyte apoptosis and liver fibrotic responses [19]. Engulfment of apoptotic bodies by hepatic stellate cells (HSCs) stimulates fibrogenic activity [20]; and the DNA from apoptotic hepatocytes can act as an important mediator of HSC activation and differentiation [21].

The* TGF-β* cytokine is involved in cell survival, proliferation, differentiation, and angiogenesis [13, 14].* TGF-β* binding to its receptor causes recruitment and phosphorylation of other TGF-*β* receptors that could activate the Smad pathways. The initiation of* TGF-β/Smad* signaling pathway started by the formation of heteromeric receptor complexes [22, 23] that lead to phosphorylation of* Smad-2* and* Smad*-*3* and then the formation of a complex with Smad4. The phosphorylated* Smad-2* and* Smad-3* associate with* Smad-4* and then enter the nucleus to regulate gene transcription [17]. Furthermore, Smad proteins are mediators for the* TGF-β*-induced apoptosis [24]. Transforming growth factor-*β*1 is formed mainly by fibroblast and contributes to fibrosis development, hypertrophy, and apoptosis [25]. The role of* TGF-β/Smad* signaling in hepatocytes in the development of NASH is not well understood and its role in metabolic disease is still limited. In liver,* TGF-β* signaling participates in fibrogenic response through hepatic stellate cell activation [26]. In chronic liver diseases, HSCs are primary target for active* TGF-β*, and thus it plays a role in progression of fibrosis in advanced NAFLD. Procollagen-*α*_1_ and procollagen-*α*_2_, tissue inhibitor of metalloproteinase-1 and metalloproteinase-2 (TIMP-1 and TIMP-2), and plasminogen activator inhibitor-1 (PAI-1) are identified as direct* TGF-β* target genes in HSCs [27–29]. However, the transcriptional activation of myofibroblast markers *α*-smooth muscle actin (*SMA*) and connective tissue growth factor (*CTGF*) is induced in a TGF-*β*-independent manner. Instead,* TGF-β* signaling is required for *α-SMA* organization and stress-fiber formation [30].

Heme oxygenase-1 (*HO-1*) has protective activity against acute and chronic liver injury [31] and is regulated by* Nrf2* transcription factor [32]. Studies found that during hepatic injury, induced by oxidative stress,* HO-1* and* Nrf2* were downregulated and were associated with* NF-κB* upregulation [33, 34]. The Rutin administration results in* Nrf2*,* HO-1*, and* NF-κB* overexpression. Rutin acts as HO-1 inducer in liver ischemia-reperfusion injury rat model [35]. The nuclear translocation of HO-1 could regulate the genes responsible for cytoprotection against oxidative stress [36]. The release of ROS is known to activate inhibitory kappa-B kinase which causes phosphorylation of* IκB*. Release of free* NF-κB* enhances the inflammatory cytokines [37] and suppresses the antioxidant genes by downregulating* Nrf-2/HO-1* pathway. Rutin is capable of inhibiting* NF-κB* and activating the* Nrf-2* pathway. Sirtuin 1 (*Sirt1*), a member of sirtuin proteins family, downregulation is associated with high insulin resistance and loss in mitochondrial biogenesis cells [38]. High-cholesterol diet decreases genes expression levels that are involved in muscle mitochondrial biogenesis and function such as* Sirt1*, peroxisome proliferator-activated receptor coactivator-1 (*PGC-1*), and mitochondrial transcription factor A (*Tfam*) [39]. Rutin increases the expression levels of* Sirt1, PGC-1, and Tfam* in skeletal muscle and brain of mice, which lead to increase in muscle mitochondrial biogenesis and function [40]. Therefore the antiobesity property of Rutin might be associated with Rutin-mediated muscle mitochondrial changes.

Flavonoids are polyphenolic compounds found in plants and have an important role in detoxification of free radicals [41]. Rutin is a flavonoid glycoside that possessed different protective effects [42, 43] against lipid peroxidation and oxidative-stress-mediated diseases [44]. Therefore, the present study was aimed to investigate the preventive effect of Rutin against HCD-induced hepatotoxicity in rats through studying genes expression in the* TGF-β/Smad* pathways.

## 2. Materials and Methods

### 2.1. Animals

Forty male Wistar albino rats weighing between 80 and 180 g were obtained from the Animal Care Center, College of Pharmacy, King Saud University, Riyadh, Saudi Arabia. The animals were acclimatized to laboratory condition ten days prior to the experiment. They were fed on Purina rat chow diet (manufactured by Grain Silos & Flour Mills Organization, Riyadh, Saudi Arabia) and water on a free access basis and were maintained under standard conditions of temperature (22 ± 1°C), humidity (50–55%), and 12 h light/dark cycles. All methods including euthanasia procedure were conducted in accordance with guide for care and use of laboratory animals, Institute for Laboratory Animal Research, National Institute of Health (NIH publication number 80-23; 1996), and they have been approved by Research Ethics Committee of Excremental Animal Care Center, College of Pharmacy, King Saud University, Riyadh, Saudi Arabia.

### 2.2. Diets

Diets were prepared in pellet form by adding 0.2% Rutin (RT) (powder, Sigma, USA) or 1% cholesterol + 0.5% cholic acid (HCD) or 0.2% RT + 1% cholesterol + 0.5% cholic acid (RT + HCD) in rat chow powder. Rat chow was used as normal diets and was prepared weekly and shade dried. Food intake g/day was calculated daily for each group to the end of experiments.

### 2.3. Experimental Design

The animals were randomly divided into 4 groups of 10 rats in each as follows: Group I, control; Group II, RT; Group III, HCD; and Group IV, RT + HCD. The experimental diets were fed on a free access basis for 6 consecutive weeks. At end of the experiment, rats were weighed and sacrificed by decapitation and the trunk blood was collected in heparinized tubes. Rats livers were rapidly excised, weighed, and kept at −80°C till analysis. Plasma samples were collected after centrifugation and stored at −20°C till analysis.

### 2.4. Bioassay Measurements

Plasma levels of ALT, AST, TG, TC, HDL, and LDL were estimated by using commercially available diagnostic kits (Human, Wiesbaden, Germany).

### 2.5. Measurement of Caspase-3 Activity in the Liver Tissues

The caspase-3 activity was measured in liver tissues by using colorimetric assay ab39401 (Abcam, Cambridge, MA 02139-1517, USA) kit according to the manufacturer instructions. In brief, 0.2 mg of liver tissues was not homogenized completely in 50 *μ*L cell lysis buffer on ice for 10 minutes. After centrifugation, the protein concentrations were adjusted to 50–200 *μ*g protein per reaction. Fifty microlitres of caspase reaction mix containing 10 mM DTT was added to each well. Two hundred micromolars of DEVD-*p*-NA substrate was added, and then after 120 min incubation at 37°C the plate was measured at 405 nm. Fold increase in caspase-3 activity can be determined by comparing sample (treated) results with the level of the untreated control.

### 2.6. Quantitative Real-Time Polymerase Chain Reaction (qRT-PCR)

Gene expression levels of* TGF-β*,* Smad-2*,* Smad-4*, caspase-3,* P21*,* P53*,* IL-3*, and* IL-6* were detected in liver tissues by quantitative real-time PCR. Total RNA were extracted from liver tissues using RNA Mini Kit (Bioline, Taunton, USA) according to the manufacturer's protocol. The quantity and integrity of total RNA were characterized using a UV spectrophotometer (Nanodrop 8000, Thermo Scientific, USA) and ethidium bromide stained agarose gel. The isolated RNA has an A 260/280 ratio of 1.9–2.0. First-strand cDNA was synthesized from 1 *μ*g of total RNA by reverse transcription with a SuperScript*™* first-strand synthesis system kit (Invitrogen, CA, USA), according to the manufacturer's instructions. Quantitative real-time PCR using ΔΔCT method was done according to our previous study [10]. *β-actin* gene is used as internal control. All primers used in this study were listed in Table 1.

### 2.7. Statistical Analysis

Differences between obtained values (mean ± SEM, *n* = 10) were carried out by one way analysis of variance followed by the Tukey-Kramer multiple comparison. The differences were considered statistically significant at *P* < 0.05.

## 3. Results

**Figure 1:**
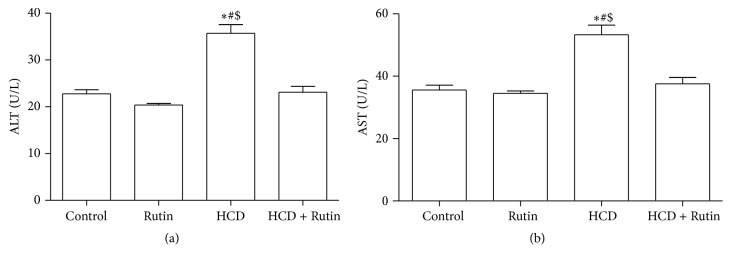
The effect of HCD, Rutin, and their combination on the plasma levels of ALT (a) and AST (b) in rats. Data are presented as mean ± SEM (*n* = 10). *∗*, #, and $ indicate significant change from control, Rutin, and HCD + Rutin, respectively, at *P* < 0.05 using ANOVA followed by Tukey-Kramer as a post-ANOVA test.

As in our previous study [10], the liver enzymes, ALT and AST plasma levels, were used as biochemical markers for early acute hepatotoxicity. Rats fed with HCD for 6 weeks had significant increase in ALT (Figure 1(a)) and AST (Figure 1(b)) levels (*P* < 0.001) compared to control and HCD + Rutin groups. Rutin supplementation alone showed no significant changes in biochemical markers. However, administration of Rutin in combination with HCD resulted in reversal of hepatic damage biomarker induced by HCD to the values as in control group. These reversal changes were significant compared to HCD group.

**Figure 2:**
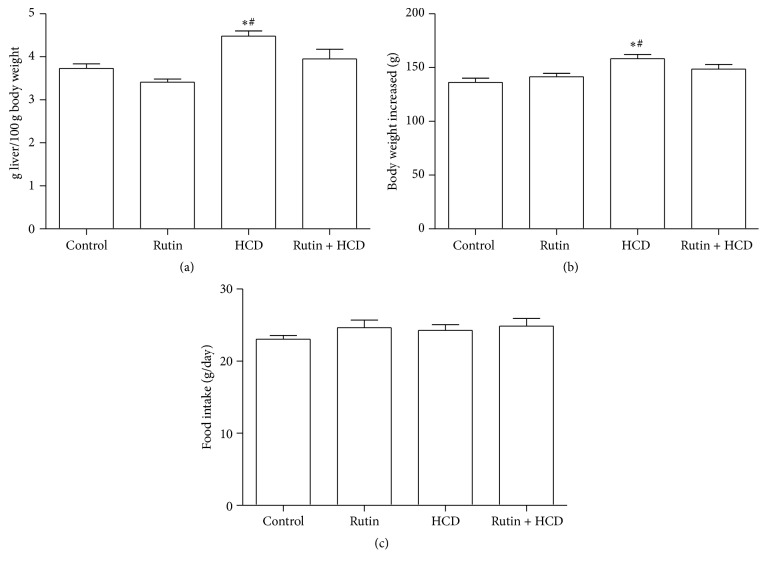
The effect of HCD, Rutin, and their combination on the liver weight (a), body weight (b), and food intake (c) in rats. Data are expressed as mean ± SEM (*n* = 10). *∗* and # indicate significant change from control, Rutin, respectively, at *P* < 0.05 using ANOVA followed by Tukey-Kramer as a post-ANOVA test.

The effect of HCD on the body and liver weights as well as the food intake during the experiment was shown in Figure 2. HCD significantly increased the liver weight (Figure 2(a)) and the body weight (Figure 2(b)) compared to control and Rutin groups. On the other hand there was no significant difference observed in food intake during experiment among all studied groups (Figure 2(c)). The supplementation of 0.2% Rutin or 1% cholesterol + 0.5% cholic acid (HCD) or 0.2% RT + 1% cholesterol + 0.5% cholic acid (RT + HCD) to the food did not influence the food intake among all groups.

**Figure 3:**
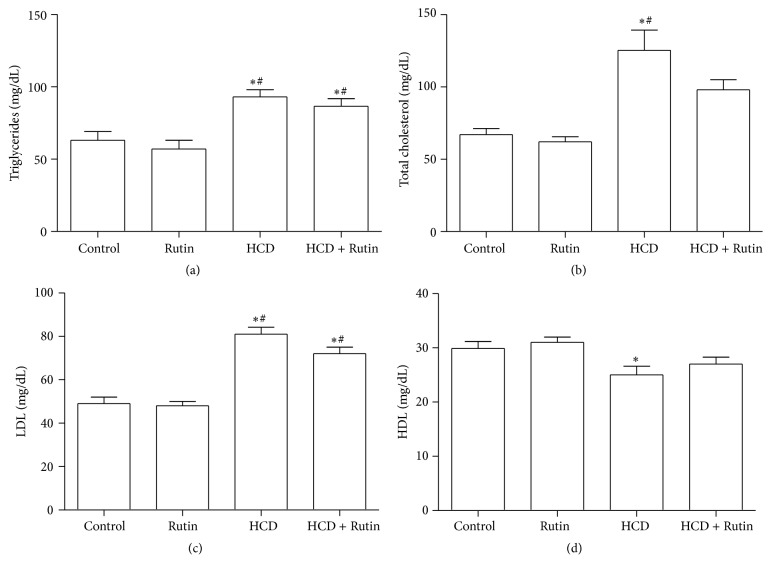
The effect of HCD, Rutin, and their combination on the plasma levels of triglyceride (a), total cholesterol (b), high-density lipoprotein (c), and low-density lipoprotein (d) in rats. Data are presented as mean ± SEM (*n* = 10). *∗* and # indicate significant change from control and Rutin groups, respectively, at *P* < 0.05 using ANOVA followed by Tukey-Kramer as a post-ANOVA test.

The effects of HCD on the lipid parameters including TG, TC, HDL, and LDL levels were shown in Figure 3. High-cholesterol diet significantly increased plasma levels of TG (Figure 3(a)), TC (Figure 3(b)), and LDL (Figure 3(c)) by 48%, 89%, and 67%, respectively, and significantly decreased the HDL (Figure 3(d)) levels by 17% compared to control group. Rutin supplementation in combination with HCD significantly increase TG, TC, and LDL levels and insignificantly decreased plasma levels of HDL compared to control group. On the other hand there were no significant differences observed in the plasma lipids levels (TG, TC, HDL, and LDL) in RT group compared to control group.

**Figure 4:**
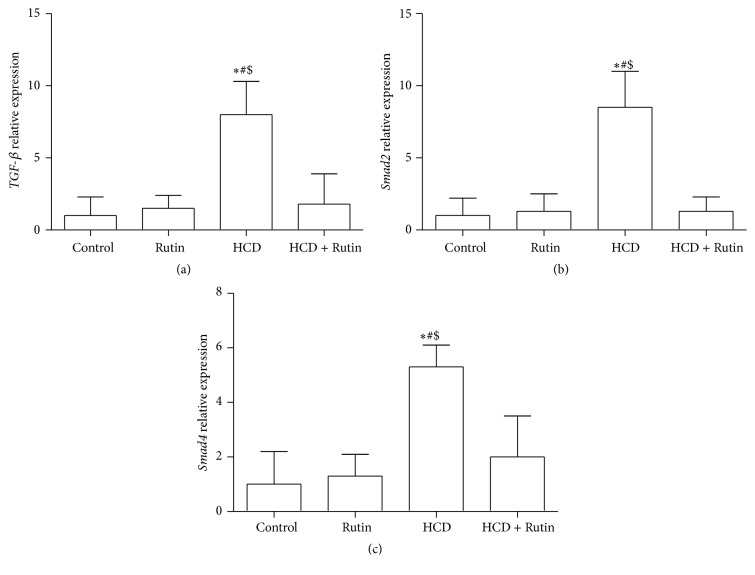
The effect of HCD, Rutin, and their combination on the levels of* TGF*-*β* (a),* Smad-2* (b), and* Smad-4* (c) expression level in rats liver tissues. Data are presented as mean ± SEM (*n* = 10). *∗*, #, and $ indicate significant change from control, Rutin, and HCD plus Rutin, respectively, at *P* < 0.05 using ANOVA followed by Tukey-Kramer as a post-ANOVA test.

The expression levels of* TGF-β, Smad-2, Samd-4, P21, caspase-3*, and* P53* genes were studied in rats hepatic cells to investigate whether HCD stimulates the* TGF-β1* signaling cascade that leads to liver fibrosis and apoptosis. The effects of HCD, Rutin, and their combination on* TGF-β1*,* Smad-2*, and* Smad-4* expression levels were shown in Figure 4. In HCD group, the expression levels of* TGF-β* (Figure 4(a)),* Smad-2* (Figure 4(b)), and* Smad-4* (Figure 4(c)) were significantly increased by 8-, 8.5-, and 5.3-fold, compared to the control group. On the other hand, administration of Rutin in combination with HCD induced a significant repair of the HCD-induced alteration in the gene expression of* TGF-β*,* Smad-2*, and* Smad-4* compared to the HCD group expression levels.

**Figure 5:**
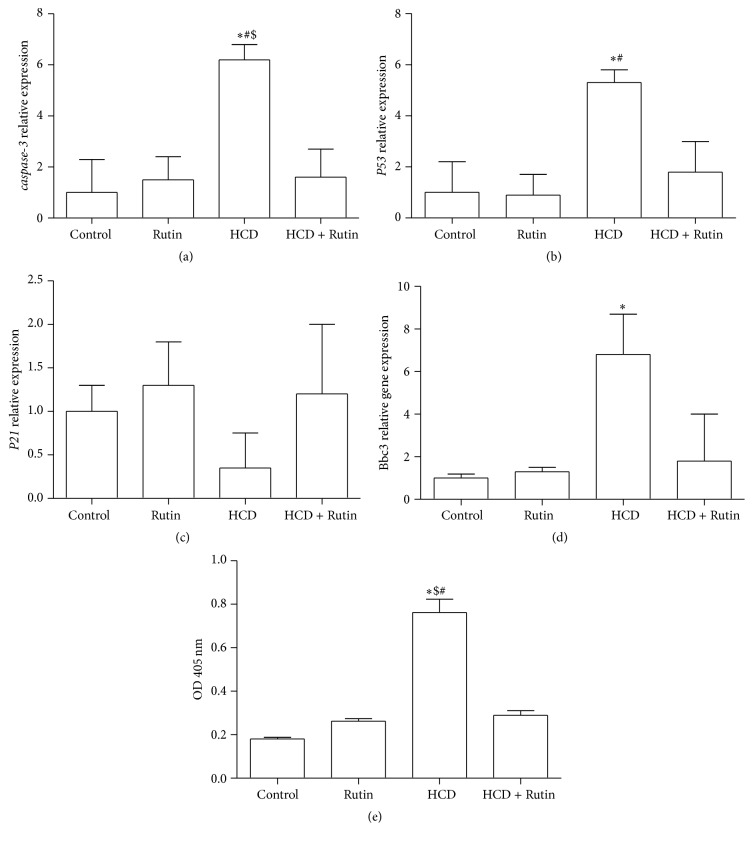
The effect of HCD, Rutin, and their combination on the expression levels of* caspase-3* (a),* P53* (b),* P21* (c), Bbc3 (d), and cleavage caspase-3 (e) in rat liver tissues. Data are presented as mean ± SEM (*n* = 10). *∗*, #, and $ indicate significant change from control, Rutin, and HCD plus Rutin, respectively, at *P* < 0.05 using ANOVA followed by Tukey-Kramer as a post-ANOVA test.

Figure 5 shows the effect of HCD, Rutin, and their combination on the expression level of* caspase-3* (a),* P53* (b),* P21* (c), and* Bbc3* (d) and on the caspase-3 activity (e) in rat liver tissues. High-cholesterol diet resulted in significant increases in expression levels of* caspase-3*,* P53*, and* Bbc3* by 6.2-, 5.3-, and 6.8-fold, respectively, compared to the control group. Interestingly, administration of Rutin in combination with HCD resulted in a complete reversal change of* caspase-3*,* P53*, and* Bbc3* induced by HCD to the normal expression levels as in control group. The activity of caspase-3 cleavage in liver tissues of control rats which feed on rat chow was hardly detectable, while a significant increase by 289% and 192% in the levels of cleavage caspase-3 was observed in the HCD-fed rat compared to control and Rutin group, respectively. The Rutin in combination with HCD significantly decreased the levels of cleavage caspase-3 by 62% compared to HCD group. On the other hand, HCD suppressed the expression level of* P21* by 2.85-fold, while administration of Rutin in combination with HCD resulted in a complete reversal change of* P21* induced by HCD to values as in control group.

**Figure 6:**
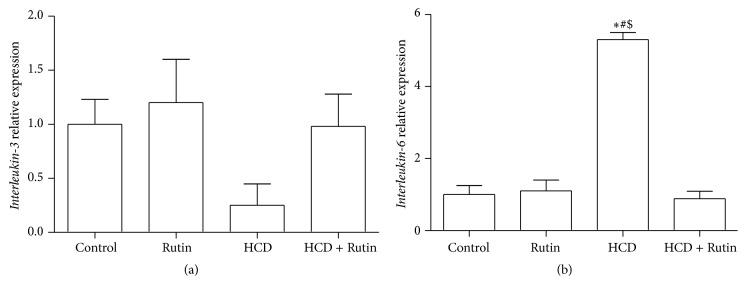
The effect of HCD, Rutin, and their combination on the expression levels of* Interleukin-3* (a) and* Interleukin-6* (b) in rat liver tissues. Data are presented as mean ± SEM (*n* = 10). *∗*, #, and $ indicate significant change from control, Rutin, and HCD plus Rutin, respectively, at *P* < 0.05 using ANOVA followed by Tukey-Kramer as a post-ANOVA test.

Figure 6 shows the effects of HCD, Rutin, and their combination on the expression level of* IL-3* (a) and* IL-6* (b) in rat liver tissues. The expression level of* IL-6* was significantly increased by 5.8-fold in rats fed with HCD compared to control group. On the other hand, rats fed with HCD had decreases in the expression levels of* IL-3* by 3-fold compared to control group. Interestingly, supplementation of Rutin in combination with HCD resulted in a complete reversal change of* IL-6* and* IL-3* induced by HCD to the normal values as in control group.

## 4. Discussion

Obesity, hypertriglyceridemia, and/or hypercholesterolemia are the common causes for many diseases such as cardiovascular [45] and liver diseases [46, 47]. Rat fed with HCD can be used as model of the human obesity syndrome [48]. In liver disease, cell repair, inflammation, regeneration, and fibrosis may all be triggered by apoptosis [13, 14]. Nonalcoholic fatty liver disease can end in cirrhosis and hepatocellular carcinoma [6]. The present study examined the hepatoprotective effect of Rutin against hepatotoxicity induced by HCD in rat model. The HCD caused hepatotoxicity through increasing liver enzymes, ALT and AST. In agreement with earlier studies, the elevated ALT and AST levels are attributed to hepatic damage that may contribute to oxidative stress unbalance [45, 49]. Earlier it is reported that Rutin has potential to reduce the oxidative stress in liver, kidney, and brain tissues of rats [50]. As a result of Rutin supplementation, ALT and AST levels were lowered leading to decrease in the hepatic damage caused by HCD feedings. The present study showed that Rutin can protect hepatocyte against the toxicity induced by HCD.

High-cholesterol diet leads to dyslipidemic syndrome and hyperlipidemia that is characterized by increase in TG and decrease in HDL-cholesterol [51]. In the current study, high-cholesterol diet led to TG increase and HDL-cholesterol decrease. The accumulation of excess triglycerides in hepatocytes forms steatotic droplets in NAFLD [52, 53]. The hyperlipidemia, hypertriglyceridemia, and the decreasing in HDL-cholesterol increase the risk of NAFLD [54]. In the present study, Rutin supplement ameliorated the effect of HCD by lowering TC, TG, and LDL levels. Similarly, Rutin decreased the lipid in hypercholesterolemic rats by reducing the activity of 3-hydroxy-3-methyl-glutaryl-CoA reductase [55]. Also, Rutin has a strong ability to chelate multivalent metal ions, especially zinc, calcium, and iron [56].

The immunoregulatory cytokine such as IL-6 is a multifunctional cytokine that regulates some biological process and may play essential role ranging from inflammation to host defense toward tissue injury [57]. Interleukin-6 with TNF*α* stimulates hepatic lipogenesis [58], impairs insulin signaling [59], and can trigger different key steps in insulin signaling pathway [60]. In the current study, HCD increased* IL-6* expression level in NAFLD compared to control group. Similar study found that TNF-*α* and IL-6 levels were significantly increased in NAFLD patients [61]. Interleukin-6 helps the hepatic survival by stimulating liver recovery *‎*and gives hepatoprotection [62, 63]. Binding of* IL-6* to its receptor (*IL-6R*) prompts *‎*STAT3 pathway activation through binding to glycoprotein 130 (gp130). IL-6 acts as both pro- and anti-inflammatory cytokines and may mediate liver damage through different pathways. Elevated levels of* IL-6* are associated with disease states [64]. The soluble form of* IL-6R* in addition to membrane-bound receptor binds to IL-6 and prolongs its plasma half-life [65]. The soluble* IL-6R* has roles in cellular proliferation, differentiation, and activation of inflammatory responses [66, 67]. In the current study, downregulated* IL-6* expression level as a result of Rutin administration may lead to the attenuation of the diet-induced hepatotoxicity. Rutin possesses anti-inflammatory effect by downregulating* IL-6* and* TGF-β1*, which promotes extracellular matrix deposition and fibrosis [68]. It has been reported that Rutin produced anti-inflammatory effects by inhibiting proinflammatory cytokines in adjuvant-induced arthritis in rats [69].

IL-3 cytokine is important for cell survival, proliferation, and differentiation [70, 71] and participates in the response to stress [72]. IL-3 may be involved in alcohol induced liver injury with unclear role [73]. In the current study, significant reduction in* IL-3* was observed following HCD feeding. The downregulation of* IL-3*, in HCD group, suggested the potential involvement of* IL-3* in HCD-induced liver injury. Recent study reported that the downregulation in IL-3 is associated with liver injury [74]. Similarly, Sheng et al. found a decrease in level of* IL-3* in rat treated with* Dioscorea bulbifera* [74].

*TGF-β1* gene downregulation can suppress the liver fibrosis and apoptosis [75]. In the present study, HCD expresses high levels of hepatic* TGF-β1*,* Smad-2*, and* Smad-4* gene expression. These findings suggest the possible involvement of* TGF-β1*,* Smad-2, and Smad-4 *genes in the regulation of hepatotoxicity process. Phosphorylation of* Smad-2* and* Smad-3* by* TGF-β* leads to* Smad-4* translocation into nucleus. In the nucleus,* Smad-4* functions to regulate transcription of target genes including* P21* and proapoptotic Bcl-2 family [76].* TGF-β1/Smad-2* signaling is responsible for insulin regulation and pancreatic *β*-cell function [77]. The effects of* Smad *genes on obesity and type 2 diabetes are not well understood. In the current study, the HCD increased* Smad-2* and* Smad-4* expression levels. The role of* Smad-2* in liver fibrosis is less well characterized but in comparison with* Smad-3* seems to regulate a distinct set of target genes [78, 79]. The combination of Rutin with HCD has downregulated the* Smad-2* and* Smad-4* gene expression levels. These results indicated that Rutin exerted a negative regulatory effect on hepatotoxicity and fibrogenesis.* TGF-β1/Smad* pathway and* IL-6* are extensively studied as mutagenic and fibrotic factors and appear to be critical for matrix-producing cells activation.* TGF-β1* is a fibrosis mediator, which can enhance extracellular matrix deposition by activating stellate cells to turn on fibrosis gene expression and by inhibiting collagenase activity via* Smad-2/-3* signaling [26]. Thus, they might be used in liver fibrosis treatment.

Tumor suppressor gene* P53* plays a central role in fatty liver disease [80–82] and its activation may be important in fatty liver pathogenesis that facilitates apoptosis, oxidative stress, steatosis, and injury. The regulatory control of* P53* appears to fail in fatty liver disease [83] as a direct downstream target of* P53 *[84]. In the current study, HCD increases* P53* expression level. This increase in* P53 *expression level in HCD may suggest that* P53 *activation may be involved in liver injury. This finding was in agreement with another study that found a positive correlation between steatosis and* P53* expression in human liver samples [80].

Increases in the* P53* expression induce* CTGF* result in liver fibrosis in rodent and human [85]. In the current study, the increase in the expression levels of* P53* is associated with increase in the expression of* Bbc3*.* Bbc3* interacts with antiapoptotic Bcl-2 family members such as* Bcl-xL*,* Bcl-2*,* Mcl-1*,* Bcl-w*, and* A1*, inhibiting their interaction with* Bax* and* Bak* which leads to release of apoptosis-inducing factor and mitochondrial apoptotic proteins cytochrome c leading to caspase activation and cell death [86].

The stage of liver steatosis increased the expression of* P53*, in hepatocytes. Levels of* P21* often determine the cellular response to different stress induced by drug and others. In the current study, HCD induce reduction in the expression level of* P21*. Reduction of endogenous* P21* expression attenuates the growth arrest and promotes cell death. Similarly, another study reported that the reduction in the expression level of* P21* in MCF7 cells by antisense P21 RNA attenuates the growth arrest and promotes cell death [87]. Different types of cellular stress lead to induction of* P21* expression by both* P53*-dependent and -independent mechanisms. High expression of* P21*-induced growth arrest can protect cells from apoptosis. However,* P21* possesses proapoptotic functions and may play an active role in apoptosis induced by activation of members of the TNF family of death receptors. Activated* TNFα/CD95* interacts with one or several cell surface receptors to trigger caspase activation and cytochrome c release. In the current study HCD induce activation of* caspase-3*.* Caspase-3*-mediated cleavage of* P21* and subsequent upregulation of cyclin A/Cdk2 activity is considered an important mechanism for death associated with cyclin A/Cdk2 activation in different cell types.

During hepatic injury, induced by oxidative stress, the* HO-1* and* Nrf2* expression levels were downregulated and were associated with upregulation in* NF-κB* [33, 34]. Overexpression of these transcription factors acts to promote apoptosis. The Rutin administration increased* Nrf2* and* HO-1* expression levels and suppressed the expression levels of* NF-κB*. In addition to oxidative stress, high-cholesterol diet decreases the expression levels of genes involved in muscle mitochondrial biogenesis and function, such as Sirt1 [39]. The mechanisms of liver apoptosis in NAFLD induced by HCD need more study to understand the mechanism.

## 5. Conclusion

In conclusion, our findings demonstrated that Rutin could lower and attenuate hepatotoxicity induced by HCD in rat model. Signaling molecules in the* TGF-β/Smad* signaling pathway were restored to nearly their normal levels. Considering its excellent safety profile, we propose that Rutin could be used in people at high risk of developing hepatotoxicity induced by HCD.

## Figures and Tables

**Table 1:** Primer sequence used in this study.

Gene name	Forward primer	Reverse primer
*TGF-β*	5′-TGCCTGACGGTCAGGTCA-3′	5′-CAGGAAGGAAGGCTGGAG-3′
*Smad-2*	5′-CCATCCCCGAGAACACTAACTT-3′	5′-TGGTGGTCGCTAGTTTCTCCAT-3′
*Smad-4*	5′-ACCAACTTCCCCAACATTCCT-3′	5′-ACTATGGCTGCCTGCCAGAA-3′
*Caspase-3*	5′-AAATTCAAGGGACGGGTCATG-3′	5′-GAGCTTGTGCGTACAGTT-3′
*Bbc3*	5′-AGCCAAACCTGACCACTAGC-3′	5′-CCAGATGAAGGTGAGGCAGG-3′
*P53*	5′-CACCATGAGCGTTGCTCTGAT-3′	5′-GATTTCCTTCCACCCGGATAA-3′
*P21*	5′-GAGAACTGGGGAGGGCTTTC-3′	5′-TCCTGAGCCTGTTTCGTGTC-3′
*IL-3*	5′-TTGTATTCCTGCAGCTGCGA-3′	5′-GGGCTGAGGTGGTCTAGAGA-3′
*IL-6*	5′-ATCTGCCCTTCAGGAACAGC-3′	5′-AGCCTCCGACTTGTGAAGTG-3′
*β-actin*	5′-TGCCTGACGGTCAGGTCA-3′	5′-CAGGAAGGAAGGCTGGAAG-3′
